# Bioenergetic and early treatment response stratification (BIOERES): a two-variable prognostic model for early identification of treatment-resistance schizophrenia

**DOI:** 10.1038/s41398-026-03983-x

**Published:** 2026-03-31

**Authors:** Eloi Giné-Servén, Ester Boix-Quintana, Alejandro Ballesteros, Eva Daví-Loscos, Nicolau Guanyabens, Virginia Casado, Sara Silles, Alba Toll, Mattia Campana, Manuel J. Cuesta, Javier Labad

**Affiliations:** 1 Department of Psychiatry, Hospital Universitario de Navarra, Pamplona, Spain; 2 Instituto de Investigación Sanitaria de Navarra (IdiSNA), Pamplona, Spain; 3 Facultat de Medicina i Ciències de la Salut, Universitat de Barcelona (UB), Barcelona, Spain; 4 Department of Mental Health and Addictions, Hospital Universitari de Mataró, Consorci Sanitari del Maresme, Universitat Autònoma de Barcelona, Mataró, Spain; 5 Department of Neurology, Hospital Universitari de Mataró, Consorci Sanitari del Maresme, Universitat Autònoma de Barcelona, Mataró, Spain; 6 Department of General Psychiatry 2, LVR Hospital, Heinrich-Heine University, Dusseldorf, Germany; 7 Centro de Investigación Biomédica en Red de Salud Mental (CIBERSAM), Instituto de Salud Carlos III, Madrid, Spain

**Keywords:** Molecular neuroscience, Predictive markers

## Abstract

Approximately one-third of patients with first-episode schizophrenia-spectrum disorders develop treatment-resistant schizophrenia (TRS) within five years, yet reliable early predictors remain lacking. Routine cerebrospinal fluid (CSF) biomarkers may offer insights into TRS pathophysiology and enable early prognostic stratification. In this longitudinal study, we examined whether baseline CSF parameters—total protein, glucose, and lactate dehydrogenase (LDH)—predicted TRS, defined according to TRRIP consensus criteria and clozapine use. Forty-four patients with first-episode schizophrenia spectrum disorders underwent lumbar puncture during index hospitalization and were followed clinically for five years at the Mataró Mental Health Care Centre. TRS status was confirmed through detailed electronic health record review. Thirteen patients (29.5%) met TRS criteria. At baseline, these individuals had significantly lower CSF LDH concentrations compared to non-TRS patients (p = 0.014), while glucose and protein levels showed no significant differences. In adjusted logistic regression models, lower LDH remained independently associated with TRS (OR = 0.043, p = 0.031). A combined model incorporating LDH and early antipsychotic response achieved an AUC of 0.86, outperforming LDH alone (AUC = 0.73), and demonstrating good discriminative accuracy. Lower baseline CSF LDH concentrations predicted treatment resistance at five years, especially when combined with poor early antipsychotic response. This two-variable prognostic model—BIOERES (Bioenergetic and Early Response Stratification)—may facilitate early identification of high-risk patients and support personalized treatment strategies. Validation in larger, independent cohorts is needed.

## Introduction

Approximately one-third of patients experiencing a first episode schizophrenia-spectrum disorder will meet the criteria for treatment-resistant schizophrenia (TRS) within five years following the onset of the illness [1]. These patients continue to manifest persistent symptoms even after undergoing at least two trials of antipsychotic treatment, each administered at an adequate dosage and timing [2]. TRS is associated with significant functional impairment, increased healthcare utilization, societal costs, and a higher prevalence of physical health comorbidities [3]. Early identification of patients at risk for TRS is crucial to prevent delays in the initiation of evidence-based interventions and to enhance long-term clinical and functional outcomes [4]. Clozapine is the only pharmacological treatment approved for TRS; however, its prescription is frequently delayed by 5-10 years due to safety concerns and the lack of clear early diagnostic criteria for treatment resistance [5, 6]. This delay is associated with poor clinical outcomes [7]. However, early initiation of clozapine treatment may lead to a better course of illness, suggesting a potentially critical time window for its effectiveness [8]. Several variables have been associated with TRS development in first episode psychosis (FEP): younger age at onset, male sex, prolonged duration of untreated psychosis (DUP), prominent negative and cognitive symptoms, inadequate response to initial treatment, and poorer premorbid adjustment [5, 9, 10].

However, studies specifically examining early antipsychotic response as a predictor of later outcome in first-episode psychosis have yielded heterogeneous results. While some evidence suggests that limited symptom improvement during the first weeks of treatment may not reliably identify patients who will ultimately fail to respond after a full acute trial, particularly in first-episode schizophrenia [11], other studies—especially those defining early nonresponse at later time points—have reported meaningful prediction of subsequent nonresponse and nonremission [12]. This variability highlights the limitations of relying on early clinical response alone in FEP populations and underscores the need for complementary biological markers to improve early risk stratification.

The etiology of TRS remains poorly understood and is believed to involve the interaction of various environmental, genetic, and neurobiological factors [4]. It is essential to elucidate the underlying pathophysiology of TRS and identify its predictors and biomarkers for early detection and treatment [13]. In research focused on biomarkers of treatment resistance, various potential predictors have been recognized, such as plasma biomarkers of immune inflammation and metabolism [4, 14–18], neuroimaging [19], and genetic factors [20, 21].

In a previous study from our group, CSF biomarkers related to brain metabolism (LDH, glucose) were associated with key clinical variables in patients with FEP [22, 23]. Specifically, lower CSF LDH concentrations were associated with greater severity of prodromal symptoms and increased social withdrawal at illness onset, suggesting a link between reduced metabolic activity and an adverse early clinical profile. In contrast, higher LDH concentrations were predictive of poor early treatment response at two weeks. Additionally, higher CSF glucose levels were linked to more severe depressive and disorganized/concrete symptoms and to poorer symptomatic improvement over time, as well as to a higher likelihood of non-affective psychotic disorders at follow-up. Taken together, these findings support the notion that alterations in central brain metabolism are detectable at the initial stages of psychosis and may influence both short-term treatment response and long-term outcomes.

In the current study, we aimed to investigate whether routine CSF parameters (total protein, glucose, and LDH) at baseline predicted TRS during five years of follow-up after illness onset in a sample of people diagnosed with schizophrenia spectrum disorders. Based on previous data [22, 23], we hypothesized that lower baseline CSF LDH concentrations would be associated with a higher risk of developing treatment-resistant schizophrenia over the long term.

## Materials and methods

### Study design and participants

A subsample of 44 patients meeting DSM-IV criteria for a schizophrenia-spectrum disorder (schizophrenia, schizoaffective disorder, schizophreniform disorder, brief psychotic disorder, delusional disorder, psychotic not otherwise specified) were selected from our previously published original sample of 98 patients experiencing a first episode of psychosis (FEP) [23]. For this study, patients with a diagnosis of affective psychotic disorder (bipolar disorder or unipolar major depression with psychotic features) were not included in this analysis. Further inclusion and exclusion criteria are described in details in the original publication [22].

Patients were admitted to an acute inpatient unit (Adult or Child and Adolescent units from the Department of Mental Health at Hospital of Mataró, Spain) and had less than 6 weeks of antipsychotic treatment.

The study received approval from the local Ethics Committees (Hospital of Mataró, Barcelona, Spain). All participants were informed about the nature of the study and gave written informed consent for participating in the study.

### Clinical assessment

During the first week of hospital admission all patients underwent psychiatric and neurological evaluations. Diagnostic interviews were carried out by two trained psychiatrists using the Structured Clinical Interview for DSM-IV-TR (SCID-I) [24] for ≥18 years and the Schedule for Affective Disorders and Schizophrenia for school-age children, and the Present and Lifetime version (K-SADS-PL) [25] for <18 years.

The onset of prodromal and psychotic symptoms was assessed retrospectively by means of a semistructured interview with a specific ad-hoc inventory (Quick Psychosis Onset and Prodromal Symptoms Inventory [Q-POPSI]) [23]. The duration of untreated illness (DUI) and duration of untreated psychosis (DUP) were calculated. A full explanation of the Q-POPSI inventory is described elsewhere [22]. Psychopathology was assessed using three psychometric scales at admission and at 2 weeks with optimal antipsychotic treatment doses. The Positive and Negative Syndrome Scale (PANSS) [26] was used to assess positive, negative and general psychopathology symptoms. Symptoms were recoded into five subscales based on the Wallwork et al. [27] consensus: positive, negative, disorganized/concrete, excited and depressed factors. Longitudinal repeated assessments of psychopathology using the PANSS were conducted at baseline, at hospital discharge and at 5 time points over the 12-month follow-up (2, 4, 6, 9, and 12 months).

Poor early antipsychotic response was defined as a < 20% reduction in the baseline PANSS positive Wallwork factor at 2 weeks with optimal antipsychotic treatment doses. In routine clinical practice, patients showing poor early response were typically switched to an alternative antipsychotic at this time point, based on the treating clinician’s judgment. This treatment adjustment reflected usual care and was not mandated by the study protocol.

Stressful life events that occurred during the 6 months prior to admission were assessed using The List of Threatening Experiences [28].

Previous global functioning was assessed using the Global Assessment of Functioning (GAF) scale [29].

Treatment resistance within the first five years following first-episode psychosis was determined using the consensus criteria established by the Treatment Response and Resistance in Psychosis (TRRIP) working group [2]. Individuals who met these criteria were subsequently treated with clozapine. It is important to mention that, after discharge from the first-episode psychosis admission, patients continued follow-up at the outpatient unit of the Mataró Mental Health Care Centre. To confirm fulfillment of TRRIP criteria, electronic health records covering the five years following admission were thoroughly reviewed by two authors (SS and AT).

### Routine CSF studies

A lumbar puncture was performed in all participants by a neurologist, either at the emergency department before admission or at the inpatient unit during the first week of admission. All participants were studied for NMDAR-Abs and GAD65-Abs in their CSF while they participated in another study dealing with autoimmune encephalitis in psychosis [30]. None of them had a diagnosis of encephalitis. CSF samples were obtained once at baseline during index hospitalization, and repeated lumbar punctures during follow-up were not performed.

CSF was examined for blood cell counts (ref <5/µL) performed on a Sysmex XN 1000 (Sysmex Corporation, Japan) automatic counting versus manual counting chamber. Quantitative determination of glucose, total protein and lactate dehydrogenase was performed on a COBAS INTEGRA (Roche Diagnostics, Spain) using the hexokinase method (glucose), the Biuret method (total protein) and the lactate to pyruvate reaction in N-methylglucamine buffer (lactate dehydrogenase), respectively. The sensitivity of the assays was 4.35 mg/dL for glucose, 4 mg/dL for total proteins, and 10 (IU/L) for LDH. The intra-assay and interassay coefficients of variation for glucose were 0.8 and 2.5% for glucose, 2.25 and 5% for total proteins, and 1.29 and 1.7% for LDH, respectively.

### Statistical analysis

All data analyses were performed using IBM SPSS Statistics for Windows, Version 20.0 (IBM Corporation, USA) and R Statistical Software (v4.2.1; R Core Team 2022).

Total protein and LDH concentrations were natural log transformed (ln) to reduce skewness.

Student’s t test was used to compare continuous data between groups (e.g. early poor response). Statistical significance was set as a p value < 0.05 (two-tailed).

Regarding the cell count in CSF, as only 3 patients had pleocytosis ( > 5 white blood cells/μL in CSF), we decided not to explore associations between this CSF measure and treatment response in multivariate analyses. Therefore, all hypotheses on CSF variables will consider three parameters that will be treated as continuous variables (total protein, LDH and glucose).

### Resistance to antipsychotic treatment

We generated receiving operating characteristic (ROC) curves to explore the diagnostic ability of single CSF parameters. ROC curves were calculated for each CSF parameter (glucose, LDH, total protein) with the pROC R library. A violin plot was generated with the library ggplot2 R library. In this graph, median values will be represented with a line and the sex distribution represented with blue (men) and red (women) colours.

To explore whether a simple combination of clinical and biological variables could improve early prediction of treatment resistance, we conducted an exploratory stratification analysis. We used early antipsychotic non-response and cerebrospinal fluid (CSF) parameters that showed significant associations with treatment resistance in the primary analyses. For exploratory stratification purposes, optimal cut-off values for CSF biomarkers were derived using receiver operating characteristic (ROC) curve analysis. The Youden’s index (J = sensitivity + specificity − 1) was used to identify the threshold that maximized the combined sensitivity and specificity for predicting treatment-resistant schizophrenia. This approach was applied to baseline CSF LDH concentrations to facilitate clinical interpretability and to enable the construction of the BIOERES stratification model. Given the modest sample size, ROC-derived cut-offs should be considered exploratory and sample-dependent, and were not intended to define definitive diagnostic thresholds.

A combined variable was then created by crossing the binary CSF biomarker group (high vs. low) with early response status (yes vs. no), resulting in four distinct clinical-biological risk profiles. For each group, we calculated the proportion of patients who developed treatment resistance at five years. This approach aimed to evaluate whether a minimal model combining one biological and one clinical factor could meaningfully stratify TRS risk at baseline.

Binomial regression analyses were used to test the hypotheses exploring the association between CSF parameters and resistance to antipsychotic treatment (TRRIP criteria, clozapine use). In these analyses, resistance to antipsychotic treatment was considered to be the dependent variable, divided into two categories: (1) nonresistance and (2) resistance (TRRIP criteria, clozapine use). In these analyses, CSF parameters (glucose, LDH, total protein) were included as independent variables, along with other covariates (age, sex, smoking, previous GAF, duration of untreated psychosis, PANSS negative scores and early treatment response), which were selected a priori based on clinical relevance and prior evidence of association with illness severity, early response, and bioenergetic markers, rather than on univariate statistical significance.

To assess the robustness of the multivariable models, sensitivity analyses were conducted using alternative measures of symptom severity. Specifically, the main regression models were re-estimated by including PANSS positive scores or PANSS total scores instead of PANSS negative scores. These analyses were performed to evaluate whether the associations between CSF biomarkers and treatment resistance were dependent on the specific symptom dimension used to adjust for baseline clinical severity.

## Results

### Sample characteristics

Demographic, clinical and biochemical data of the sample at baseline assessment are described in Table 1. Only a small proportion ( < 10%) had abnormal CSF findings.

**Table 1:** Demographic, clinical and biochemical data of 44 patients with first episode schizophrenia spectrum disorders (SSD).

Age at onset, mean (SD), years	31.3 (15.4)
Female sex, N (%)	19 (43.2%)
Smoking, N (%)	21 (47.7%)
Cannabis use (abuse or dependence), N (%)	19 (43.2%)
Alcohol use (abuse or dependence), N (%)	11 (25.0%)
First degree family history of psychiatric disease, N (%)	19 (43.2%)
Previous life stressful events, N (%)	18 (40.9%)
Duration of psychiatric prodromal symptoms, N (%)	
No prodromal symptoms	16 (36.2%)
<1 month	2 (4.5%)
1–6 months	11 (25%)
>6 months	15 (34.1%)
Duration of untreated illness, mean (SD), days	232.9 (353.5)
Duration of untreated psychosis, mean (SD), days	43.6 (65.4)
PANSS at admission, mean (SD)	
Total score	88.1 (22.7)
PANSS positive at admission	25.1 (4.6)
PANSS negative at admission	18.9 (8.1)
PANSS general at admission	44.1 (14.4)
Wallwork factors:	
Positive factor	14.3 (3.4)
Negative factor	15.6 (7.1)
Disorganized/concrete factor	9.2 (3.4)
Excited factor	10.6 (3.8)
Depressed factor	8.8 (3.4)
Poor early antipsychotic response	9 (20.5%)
GAF, mean (SD)	
Previous 6-months	70.8 (13.1)
On admission	30.5 (7.7)
At discharge	62.1 (8.7)
Treatment resistance (clozapine use) 5-years follow-up, n (%)	13 (29.5%)
Diagnoses at 5-years follow-up, n (%)	
Schizophrenia	18 (40.9%)
Schizoaffective disorder	5 (11.4%)
Schizophreniform disorder	2 (4.5%)
Unspecified psychotic disorder	9 (20.5%)
Brief psychotic disorder	4 (9.1%)
Delusional disorder	4 (9.1%)
Others	2 (4.5%)
Cerebrospinal fluid variables at admission	
Parameters, mean (SD)	
White blood cells (cells/μL)	2.9 (2.0)
Glucose (mg/dL)	65.5 (7.4)
Total proteins (mg/dL)	28.4 (8.2)
LDH (U/L at 37 °C)	26.4 (12.8)
Abnormal cerebrospinal fluid studies, N (%)	4 (9.0%)
Pleocytosis ( > 5 white blood cells/μL in CSF)	3 (6.8%)
Increased protein concentration ( > 45 mg/dL)	1 (2.3%)

*SD* standard deviation, PANSS positive and negative syndrome scale, *GAF* global assessment of functioning.

### Clinical predictors of treatment resistance

Based on TRRIP criteria and clozapine use as a proxy for treatment resistance, 13 patients were classified as TRS and 31 as non-TRS at five-year follow-up. Patients with TRS had a longer duration of untreated illness compared to non-TRS patients (p = 0.006; see Table 2). At admission, patients with TRS had significantly higher scores on the PANSS positive, negative, and general subscales, as well as on the total score, compared to non-TRS patients (p = 0.016, p = 0.040, p = 0.005, and p = 0.002, respectively; see Table 2).

**Table 2:** Sociodemographic characteristics, baseline clinical characteristics and psychopathology measures by resistance to antipsychotic treatment.

	NonTRS (N = 31)		TRS (N = 13)				
	Mean or N	SD or %	Mean or N	SD or %	t/${X}^{2}$	p	Significant comparisons^a^
Age at onset	33.5	17.3	26.1	7.7	1.465	0.150	
Female sex	13	41.2%	6	46.1%	0.066	0.797	
Substance use last 6 months	19	61.1%	6	46.1%	0.855	0.355	
Smoking	17	54.8%	4	30.7%	2.127	0.145	
Cannabis use	14	45.2%	5	38.5%	0.168	0.682	
Alcohol use	9	29.0%	2	15.4%	1.460	0.482	
First degree family history of psychiatric disease	16	51.6%	5	38.5%	0.635	0.426	
DUI (days)	141.0	149.2	452.2	564.2	-2.881	0.006	TRS>nonTRS
DUP (days)	46.3	73.0	37.2	44.0	0.415	0.680	
Acute onset psychosis	9	29.0%	7	53.8%	2.437	0.118	
Previous life stressful events	13	41.9%	5	38.5%	0.046	0.831	
Duration of the index admission (days)	18.8	10.8	35.3	22.64	-3.283	0.002	TRS>nonTRS
Previous GAF	72.8	13.1	66.1	12.2	1.562	0.126	
GAF admission	31.3	7.4	28.4	8.2	1.175	0.247	
GAF discharge	62.9	9.6	60.4	5.6	0.865	0.392	
Prodromal symptoms	19	61.3%	9	69.2%	0.250	0.617	
PANSS Positive score	24.1	3.9	27.7	5.4	-2.508	0.016	TRS>nonTRS
PANSS Negative score	17.2	7.0	22.7	9.3	-2.116	0.040	TRS>nonTRS
PANSS General score	40.2	7.0	53.2	22.2	-2.962	0.005	TRS>nonTRS
PANSS Total score	81.5	13.3	103.6	32.2	-3.246	0.002	TRS>nonTRS
CSF total proteins	29.7	8.1	25.2	7.8	1.685	0.099	
CSF LDH	29.4	13.2	19.2	8.3	2.552	0.014	TRS<nonTRS
CSF glucose	65.1	7.8	66.4	6.4	-0.513	0.610	
Poor early antipsychotic response	3	9.7%	6	46.1%	7.490	0.006	TRS>nonTRS
Antipsychotic treatment at assessment							
Olanzapine	11	35.5%	8	61.5%	3.220	0.359	
Paliperidone	6	19.4%	2	15.4%			
Risperidone	11	35.5%	3	23.1%			
Aripiprazole	3	9,7%	0	0%			

^a^p < 0.05.

*SD* standard deviation, *DUI* duration of untreated illness, *DUP* duration of untreated psychosis, *PANSS* positive and negative syndrome scale, *YMRS* young mania rating scale, *HAM-D* hamilton depressive rating scale for depression, *GAF* global assessment of functioning, *CSF* cerebrospinal fluid, *LDH* lactate dehydrogenase.

The mean ± standard deviation duration of the index admission was 18.8 ± 10.8 days for non-TRS and 35.3 ± 22.6 days for TRS (p = 0.002).

At baseline, the mean daily doses of the first prescribed antipsychotic were olanzapine 20.8 mg/day (range 7.5–30), risperidone 5.2 mg/day (range 2–9), paliperidone 9.0 mg/day (range 6–12), and aripiprazole 21.7 mg/day (range 15–30). Baseline CSF biomarkers did not differ according to the type of first prescribed antipsychotic. One-way ANOVA showed no significant differences across antipsychotic groups (olanzapine, risperidone, paliperidone, aripiprazole) for CSF LDH concentrations (F = 0.11, p = 0.95), total CSF protein (F = 1.50, p = 0.23), or CSF glucose levels (F = 0.79, p = 0.51).

### CSF biomarkers and treatment resistance

**Fig. 1:**
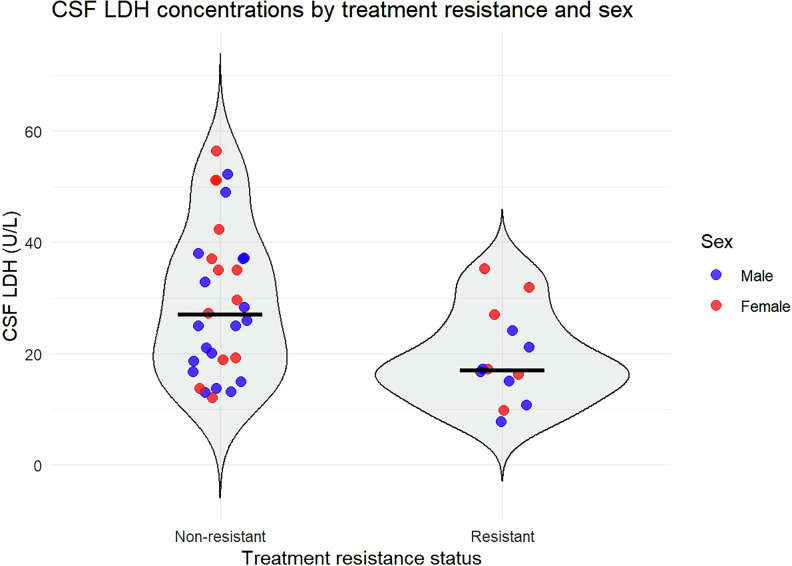
Baseline CSF LDH concentrations in TRS vs. non-TRS patients at five-year follow-up. Violin plot showing cerebrospinal fluid (CSF) lactate dehydrogenase (LDH) concentrations at baseline in patients who developed treatment resistance (TRS; n = 13) and those who did not (non-TRS; n = 31) at five-year follow-up. Patients with TRS had significantly lower baseline CSF LDH concentrations (p = 0.014). Median values are shown with horizontal lines; sex is indicated by color (blue = male, red = female).

TRS at the five-years follow-up had lower LDH baseline concentrations in CSF than non-TRS responders (Table 2; Fig. 1). There were no significant differences in other CSF parameter concentrations between these groups (p > 0.05). The ROC curves showed an area under the curve (AUC) of 0.73 for CSF LDH, 0.65 for CSF total proteins, 0.55 for CSF glucose (Figure S1).

### Combined model: LDH and early treatment response

In the exploratory combined analysis testing whether the inclusion of early antipsychotic response alongside CSF LDH would improve the predictive accuracy for treatment resistance, a logistic regression model including both variables yielded AUC of 0.86 (Figure S2), improving the AUC of LDH alone (0.73). The optimal cut-off for the combined model (probability ≥ 0.475) provided a sensitivity of 61.5% and a specificity of 96.8%, suggesting that this minimal two-variable model offers good discriminative performance for identifying patients at high risk of TRS. Using the ROC-derived cut-off of 18 U/L for CSF LDH, we generated a combined clinical-biomarker stratification (Table 3). Patients with low LDH ( ≤ 18 U/L) and poor early antipsychotic response had the highest risk of TRS (100%), followed by those with low LDH but early response (41.7%). In contrast, patients with high LDH ( > 18 U/L) and early response had the lowest TRS risk (8.7%). This stratification illustrates a clinically meaningful gradient of risk based on two easily accessible baseline variables.

**Table 3:** Risk of treatment resistance by combined LDH and early antipsychotic response groups.

CSF LDH group	Early antipsychotic response	N (total)	N (TRS)	Risk of TRS (%)
High ( > 18 U/L)	No	6	3	50.0%
High ( > 18 U/L)	Yes	23	2	8.7%
Low ( ≤ 18 U/L)	No	3	3	100.0%
Low ( ≤ 18 U/L)	Yes	12	5	41.7%

LDH group was classified using the ROC-derived cut-off value of 18 U/L. Early antipsychotic response was based on <20% reduction in PANSS positive scores at 2 weeks. Groups represent combinations of baseline CSF LDH levels and early treatment response status. TRS was defined by TRRIP criteria and clozapine use.

*CSF* cerebrospinal fluid, *LDH* lactate dehydrogenase, *TRS* treatment-resistant schizophrenia.

### Multivariable logistic regression analysis

In the binomial logistic regression analyses adjusted for age, sex, smoking, poor early antipsychotic response, previous GAF, PANSS negative score, and DUP and when considering all three CSF parameters as independent variables, LDH concentrations in CSF were inversely associated with a higher risk of TRS at the five-years follow-up (which suggests that lower baseline LDH concentrations at baseline were found in the TRS group). The results of this analysis are described in Table 4.

**Table 4:** Results of the logistic regression exploring treatment resistance at 5 years.

Variable	OR	p value	95% CI (Lower)	95% CI (Upper)
CSF total protein (ln)	0.229	0.596	0.001	53.232
CSF LDH (ln)	0.029	0.029	0.001	0.699
CSF glucose	1.129	0.256	0.916	1.391
Female sex	1.198	0.902	0.067	21.430
Age	0.945	0.267	0.855	1.044
Smoking status	0.164	0.148	0.014	1.899
Duration of untreated psychosis	0.992	0.624	0.959	1.025
Early antipsychotic non-response	23.956	0.042	1.128	508.539
Previous GAF score	0.908	0.056	0.822	1.003
PANSS negative score at admission	0.965	0.673	0.817	1.140

The Nagelkerke R^2^ of the equation was 0.645

*CSF* cerebrospinal fluid, *LDH* lactate dehydrogenase, *GAF* global assessment of functioning, *PANSS* positive and negative syndrome scale.

Sensitivity analyses replacing PANSS negative scores with PANSS positive scores or PANSS total scores yielded comparable effect estimates for baseline CSF LDH concentrations and early antipsychotic response. In all models, lower CSF LDH remained significantly associated with increased risk of treatment-resistant schizophrenia (see Supplementary Tables S1 and S2).

## Discussion

In our study, which explored whether routine CSF parameters are associated with treatment resistance at five-year follow-up after FEP, we found that lower baseline CSF LDH concentrations predicted clozapine use over the follow-up period. Furthermore, ROC curve analysis showed that CSF LDH had an AUC of 0.73, indicating a fair discriminative ability to distinguish patients who would later develop treatment resistance. This finding supports the potential utility of LDH as a prognostic biomarker in clinical settings, although further validation in larger cohorts is needed.

The observed five-year rate of treatment resistance (29.5%) is consistent with prior epidemiological data indicating that approximately 23–35% of FEP individuals develop TRS [1, 31]. The association between lower LDH concentrations in the CSF and treatment resistance, as well as previous findings of our group relating lower CSF LDH to prodromal symptoms and social withdrawal [20], supports the hypothesis that bioenergetic dysfunction plays a role in the pathophysiology and prognosis of early psychosis.

To date, this association between lower CSF LDH concentrations and early clinical features such as prodromal symptoms and social withdrawal has not been systematically examined in independent FEP samples. As such, our findings should be considered preliminary and underscore the importance of replication in larger and independent cohorts to determine the generalizability of CSF LDH as a prognostic biomarker.

Our findings should be interpreted in the context of prior results from our group showing that higher CSF LDH concentrations were associated with poorer early antipsychotic response at two weeks. Taken together, these observations suggest that LDH may function as a dynamic, stage-dependent marker of bioenergetic regulation rather than a static indicator of illness severity. Elevated LDH levels in the very early phase of treatment may reflect an acute metabolic or inflammatory stress response associated with short-term treatment resistance, whereas lower baseline LDH concentrations may indicate a more fundamental bioenergetic hypofunction, conferring vulnerability to long-term treatment resistance and TRS.

This interpretation is further supported by experimental evidence indicating that lactate-related alterations in schizophrenia are heterogeneous and depend on illness stage and cell-type–specific mechanisms. In particular, preclinical models with primary astrocytic dysfunction, such as astrocyte-specific DISC1 models, have shown reduced lactate availability, consistent with impaired astrocyte–neuron lactate shuttling [32]. These findings provide a mechanistic framework through which lower CSF LDH concentrations in first-episode patients may reflect reduced metabolic flexibility rather than compensatory increases in glycolytic activity and lactate production more commonly described in chronic stages of schizophrenia.

Importantly, our study identified a simple two-variable model—combining baseline CSF LDH and early antipsychotic response—that achieved high predictive accuracy for TRS (AUC = 0.86). We have termed this model BIOERES (Bioenergetic and Early Response Stratification), reflecting its integration of a biological marker of brain energetics and an early clinical response measure. Patients with both low LDH and poor early response had a 100% TRS rate in this cohort, whereas those with high LDH and good response showed a risk below 10%. This clinically intuitive stratification suggests that combining biological and early clinical data could meaningfully guide early treatment decisions, such as closer monitoring, early clozapine consideration, or adjunctive interventions in high-risk patients. Moreover, recent findings indicate that clozapine exposure itself may reduce leukocyte mtDNA copy number [33], underscoring how mitochondrial vulnerability could intersect with glycolytic shifts reflected by CSF LDH. These observations highlight the relevance of integrated bioenergetic markers in psychosis and their potential role in predictive models of treatment resistance.

LDH is a ubiquitous enzyme that catalyses the interconversion of pyruvate and lactate, representing a key node between glycolysis and oxidative metabolism [34]. There is increasing evidence that schizophrenia involves widespread disruptions in brain energy metabolism, including abnormalities in glycolysis, the tricarboxylic acid (TCA) cycle, and oxidative phosphorylation [35]. Postmortem studies have demonstrated LDH complex (LDHA/B) dysregulation in the anterior cingulate cortex, corpus callosum, and hippocampus [36], and abnormal lactate metabolism has been identified through metabolic imaging techniques [35]. In line with this, Henkel et al. [36] recently proposed that mitochondrial dysfunction and impaired glucose utilization are core features of schizophrenia pathophysiology, affecting the energy supply of fast-spiking GABAergic interneurons and contributing to excitation–inhibition imbalance in prefrontal circuits. This mechanism may underlie persistent negative symptoms and cognitive impairment, and could be reflected in peripheral or CSF-based bioenergetic markers [37].

Interestingly, experimental modulation of LDH activity has shown therapeutic effects in other neurological conditions, such as temporal lobe epilepsy, where LDH inhibition via pharmacological agents (e.g., oxamate, stiripentol, isosafrole) or ketogenic diet (KD) interventions suppresses spontaneous recurrent seizures in animal models [38], and KD improves seizure control in patients with refractory epilepsy [39]. Although the KD has been poorly studied in schizophrenia, preliminary evidence from clinical case reports and a recent single-arm clinical trial suggests potential psychiatric symptom improvement [40, 41]. Our finding that lower CSF LDH concentrations predict treatment resistance aligns with these models of metabolic vulnerability and supports the idea that targeting bioenergetic pathways may offer novel opportunities for therapeutic intervention in TRS.

Our study had several limitations that need to be acknowledged. First, the original project was initially designed to study autoimmunity in the CSF and serum, and our study is a secondary analysis, as we had available information on routine CSF biomarkers that was not explored in our previous study [30]. However, the original project was not designed to control for factors that could affect the bioenergetic system (such as assessing dietary habits or performing lumbar puncture under fasting conditions).

In addition, CSF biomarkers were assessed only once at baseline during index hospitalization, and repeated lumbar punctures during follow-up were not performed. As a result, we were unable to examine longitudinal changes in CSF LDH over time or to assess whether bioenergetic alterations evolve in parallel with clinical course or treatment exposure.

Second, the patients with FEP were receiving antipsychotic treatment and were not drug naïve. However, we aimed to reduce any long-term treatment effects by excluding patients who had received antipsychotic treatment for longer than 6 weeks.

Third, although the original cohort included 98 patients with first-episode psychosis, we restricted the present analysis to a subsample of 44 individuals diagnosed with schizophrenia spectrum disorders. This decision was made to enhance diagnostic and treatment-response homogeneity, as the inclusion of affective psychosis may have introduced additional variability in clinical course and pharmacological response. While this improves internal validity, it may limit the generalizability of our findings to broader FEP populations.

Fourth, treatment resistance was modeled as a binary outcome at five-year follow-up, and the timing of transition to TRS was not incorporated into the regression models. This approach was chosen given the limited sample size and incomplete availability of precise dates for meeting TRS criteria, but it precluded formal time-to-event analyses. Future studies with larger samples and detailed longitudinal treatment data should examine whether CSF LDH and early treatment response predict not only the occurrence but also the timing of treatment resistance.

In addition, some of the BIOERES stratification groups included a small number of participants, particularly the low CSF LDH/no early antipsychotic response stratum. As a result, these subgroup estimates should be interpreted with caution and considered exploratory. Replication in larger samples will be necessary to confirm the robustness and clinical utility of this stratification approach.

Further prospective validation in larger and independent samples is warranted. In particular, future studies should explore the applicability of the BIOERES model in multicenter cohorts or existing datasets that include CSF biomarkers and standardized measures of early antipsychotic response. Cross-cohort replication will be key to determine the generalizability, clinical utility, and potential integration of this stratification approach into real-world early intervention programs.

In conclusion, our findings suggest that baseline CSF LDH concentrations, particularly when combined with early antipsychotic response, may serve as an early prognostic tool for treatment resistance. This supports the broader hypothesis that bioenergetic dysfunction contributes to TRS pathophysiology, and opens avenues for targeted early intervention strategies. Further prospective validation in larger and independent samples is warranted.

## Supplementary information

Table S1

Table S2

Figure S1

Figure S2

## Data Availability

The datasets generated and analysed during the current study are not publicly available owing to participant confidentiality but are available from the corresponding author upon reasonable request.
