# Supplementary material for: Bioenergetic and early treatment response stratification (BIOERES): a two-variable prognostic model for early identification of treatment-resistance schizophrenia
Source: Transl Psychiatry. 2026 Mar 31;16:220. doi: 10.1038/s41398-026-03983-x (PMC13040051; doi:10.1038/s41398-026-03983-x)
Supplement: Supplementary file 1 — Table S1 [file 41398_2026_3983_MOESM1_ESM.docx]

**Table S1. Results of the logistic regression exploring treatment resistance at 5 years (adjusted for PANSS positive score)**

| **Variable** | **OR** | **p value** | **95% CI (Lower)** | **95% CI (Upper)** |
| --- | --- | --- | --- | --- |
| CSF total protein (ln) | 0.239 | 0.590 | 0.001 | 43.637 |
| CSF LDH (ln) | 0.025 | 0.037 | 0.001 | 0.793 |
| CSF glucose | 1.124 | 0.277 | 0.910 | 1.389 |
| Female sex | 0.961 | 0.978 | 0.059 | 15.636 |
| Age | 0.964 | 0.508 | 0.866 | 1.074 |
| Smoking status | 0.263 | 0.298 | 0.021 | 3.262 |
| Duration of untreated psychosis | 0.992 | 0.610 | 0.962 | 1.023 |
| Early antipsychotic non-response | 21.535 | 0.048 | 1.034 | 448.471 |
| Previous GAF | 0.914 | 0.061 | 0.832 | 1.004 |
| PANSS positive score | 1.116 | 0.329 | 0.895 | 1.391 |

The Nagelkerke R^2^ of the equation was 0.659

Abbreviation: CSF, cerebrospinal fluid; LDH, lactate dehydrogenase; GAF, Global Assessment of Functioning; PANSS, Positive and Negative Syndrome Scale.
