# Supplementary material for: Bioenergetic and early treatment response stratification (BIOERES): a two-variable prognostic model for early identification of treatment-resistance schizophrenia
Source: Transl Psychiatry. 2026 Mar 31;16:220. doi: 10.1038/s41398-026-03983-x (PMC13040051; doi:10.1038/s41398-026-03983-x)
Supplement: Supplementary file 2 — Table S2 [file 41398_2026_3983_MOESM2_ESM.docx]

**Table S2. Results of the logistic regression exploring treatment resistance at 5 years (adjusted for PANSS total score)**

| **Variable** | **OR** | **p value** | **95% CI (Lower)** | **95% CI (Upper)** |
| --- | --- | --- | --- | --- |
| CSF total protein (ln) | 0.396 | 0.741 | 0.002 | 95.659 |
| CSF LDH (ln) | 0.013 | 0.029 | 0.000 | 0.639 |
| CSF glucose | 1.125 | 0.287 | 0.906 | 1.398 |
| Female sex | 0.671 | 0.790 | 0.035 | 12.733 |
| Age | 0.972 | 0.590 | 0.875 | 1.079 |
| Smoking status | 0.357 | 0.433 | 0.027 | 4.699 |
| Duration of untreated psychosis | 0.995 | 0.733 | 0.964 | 1.026 |
| Early antipsychotic non-response | 16.457 | 0.086 | 0.676 | 400.828 |
| Previous GAF | 0.922 | 0.110 | 0.834 | 1.019 |
| PANSS total score | 1.050 | 0.220 | 0.971 | 1.135 |

The Nagelkerke R^2^ of the equation was 0.681

Abbreviation: CSF, cerebrospinal fluid; LDH, lactate dehydrogenase; GAF, Global Assessment of Functioning; PANSS, Positive and Negative Syndrome Scale.
