# Supplementary figures and images for: Bioenergetic and early treatment response stratification (BIOERES): a two-variable prognostic model for early identification of treatment-resistance schizophrenia
Source: Transl Psychiatry. 2026 Mar 31;16:220. doi: 10.1038/s41398-026-03983-x (PMC13040051; doi:10.1038/s41398-026-03983-x)

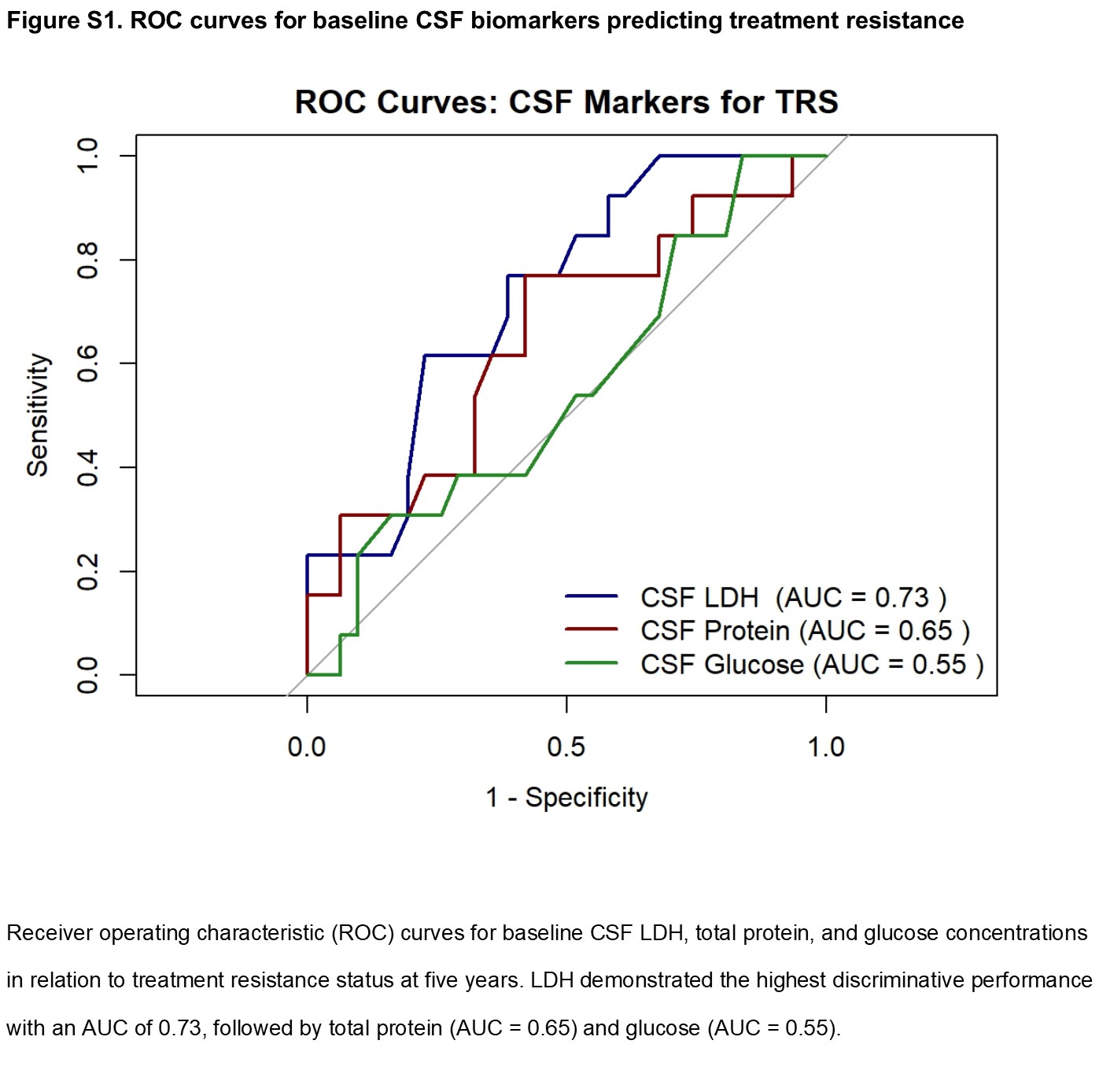

Supplement: Supplementary file 3 — Figure S1 [file 41398_2026_3983_MOESM3_ESM.jpg]

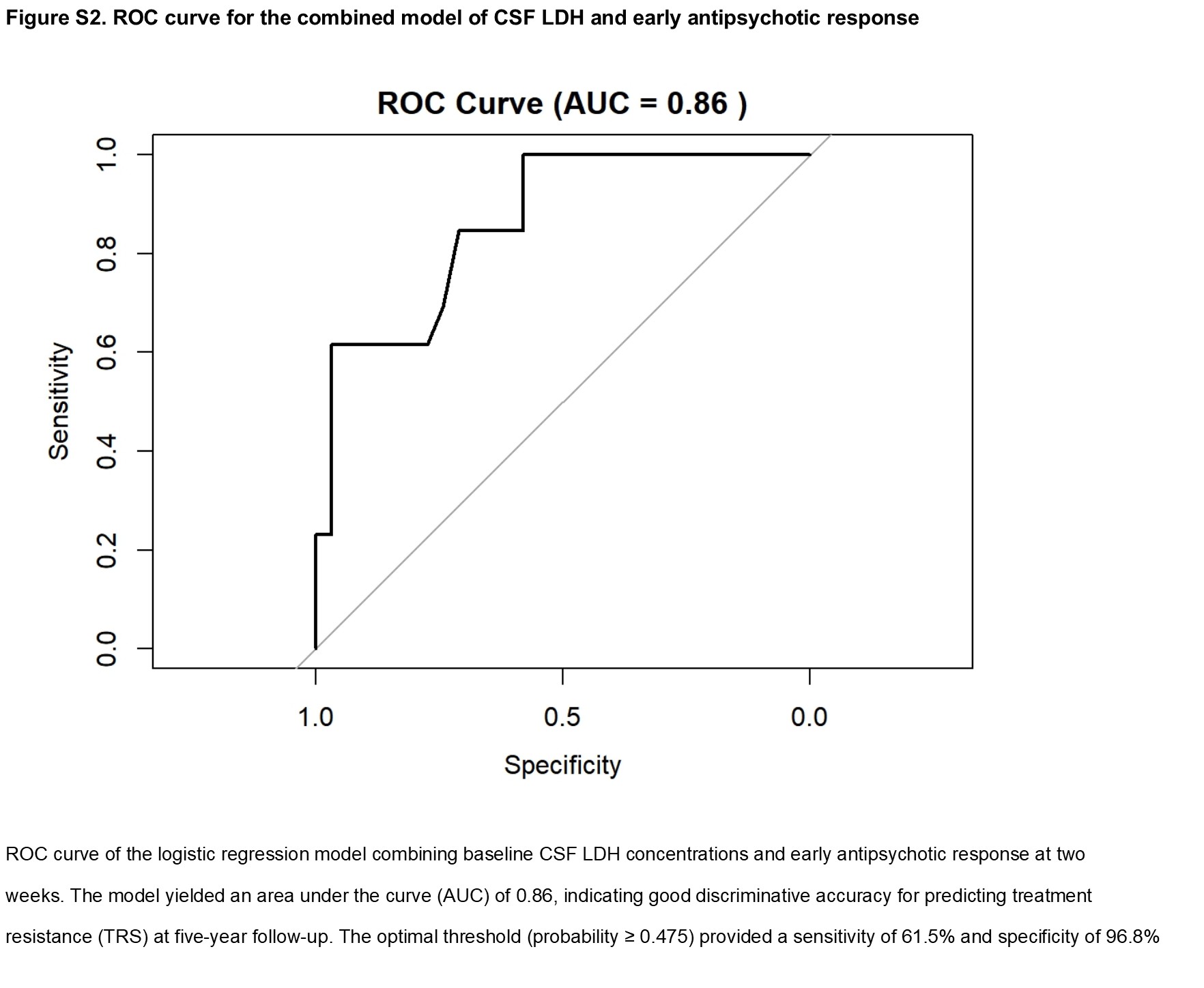

Supplement: Supplementary file 4 — Figure S2 [file 41398_2026_3983_MOESM4_ESM.jpg]
